# Supplementary material for: Does digital transformation enhance the core competitiveness?—Quasi-natural experimental evidence from Chinese traditional manufacturing
Source: PLoS One. 2023 Nov 30;18(11):e0289278. doi: 10.1371/journal.pone.0289278 (PMC10688857; doi:10.1371/journal.pone.0289278)
Supplement: Attachment — Submitted filename: Response to Reviewers.doc [file pone.0289278.s001.doc]

**Response Letter**

Dear Dr.Syed Abdul Rehman Khan

Re: Manuscript ID: PONE-D-23-15731 and Title: Does digital transformation enhance the core competitiveness?--evidence from Chinese traditional manufacturing

Thank you for editor’ and reviewers’ opinions, these comments are very helpful to improve the quality of the manuscript. We have carefully revised our manuscript, further clarify the logic of writing for improving the quality of the manuscript. Words in blue are the changes we have made in the manuscript. Now we response the reviewers’ comments with a point by point and highlight the changes in revised manuscript. Full details of the files are listed. We sincerely hope that you find our responses and modifications satisfactory and that the manuscript is now acceptable for publication.

Kind regards

Yours

LiuYang Zhang

**Response to reviewers**

We gratefully thank all reviewers for the time spent, making their constructive remarks and useful suggestions, which have significantly raised the quality of the manuscript and have enabled us to improve the manuscript. Each suggested revision and comment brought forward by the reviewers was accurately incorporated and considered. Modified content is highlighted in blue. Below the comments of the reviewers are response point by point and the revisions are indicated.

**Reviewer #1:**

The reviewer believes that the topic “Does digital transformation enhance the core competitiveness?--evidence from Chinese traditional manufacturing” is worthy of investigation. However, the following needs to be addressed. There are minor and major issues that should be corrected. I believe the paper could be further strengthened by added information about.

Please reorganize the manuscript at the journal request. Please change the reference format.

1.Comments:The language of this manuscript is very bad and needs help from native speakers.

1.Response: We apologize for the language problems in the original manuscript. We had found a professional polishing agency recommended by Plos One to modify and improve the expression and grammar of the language. and I hope this can help improve the language quality of the article.

2.Comments:The title of the manuscript should fully demonstrate the content of this study and the relevant subjects.

Response: Thank you for your rigorous consideration. After discussion, we decided to change the title of our paper to： Does digital transformation enhance the core competitiveness?--Quasi-natural experimental evidence from Chinese traditional manufacturing. We hope this will make the title more relevant.

3.Comments:Abstracts should include the purpose and findings of the study.

Response: We appreciate it very much for this good suggestion. we have rewritten the abstract and changed the structure of the abstract.

Abstract: :

In the era of the digital economy, building an internationally competitive manufacturing industry with intelligent manufacturing as its main focus is the only way to promote the transformation of a country into a manufacturing power and achieve high-quality economic development. To explore whether Digital transformation can improve the core competitiveness of traditional manufacturing enterprises, and what factors affect this process, this study establishes the core competitiveness system of enterprises through the principal component analysis(PCA) method and discusses the above issues through the construction of a double difference model. The results of this research from China's traditional manufacturing industry are as follows. (i) The digital transformation of enterprises has significantly improved their core competitiveness and has a certain time lag effect. (ii) In the process of enterprise digital transformation, enterprise management capabilities, environmental uncertainty, and enterprise operational efficiency will positively enhance the results of enterprise digital transformation. (iii) The enhancement of core competitiveness caused by digital transformation is more significant for the market leaders and laggards. (iv) Compared with non-state-owned enterprises, the Digital Transformationn of state-owned enterprises has a more obvious effect on promoting their core competitiveness. (v) In comparison with enterprises with low government subsidies, the Digital Transformation of enterprises with high government subsidies plays a more significant role in promoting their core competitiveness. In addition, this study proposes policy guidance and practical guidance for digital transformation to accelerate the promotion of core competitiveness of traditional manufacturing industry.

4.Comments:PARAGRAPH. Digital transformation is an important measure......... This a very vague statement. These sentences do not provide any information on how the concept could be conceptualized?

Response: Thanks for your careful check. We’ve changed [Digital transformation is an important measure........] to [Therefore, China proposes using digital transformation and digital manufacturing as the keys to achieving the high-quality development of the domestic manufacturing industry](Page 3, Line 17-19)

5.Comments:PARAGRAPH. Therefore, the discussion......... This section should explain the study's context and research objective. Furthermore, the research gap needs to be narrowed after analyzing the previous studies. The research method is not adequately explained in the first section.

-Introduction, what authors wanted to convey. Here author must build research gap following the previous studies.-The manuscript does not answer the following concerns: Why is it timeliness to explore such a study? What makes this study different from the previously published studies? Are there any similarly findings in line with the previously published studies? Are the findings different from prior academic studies that were conducted elsewhere, if any? For example, information Innovation and Innovation Ecosystems, what it requires, what are the new technologies, some recent issue highlights the importance. See the following: Enhancing Digital Innovation for the Sustainable Transformation of Manufacturing Industry: A Pressure-State-Response System Framework to Perceptions of Digital Green Innovation and Its Performance for Green and Intelligent Manufacturing

. https://doi.org/10.3390/systems10030072

Response: We agree and appreciate with the reviewer’s assessment. After carefully studying the paper you provided, we have rewritten this section, mainly summarizing the shortcomings of previous research and highlighting our own research highlights. I hope this meets your suggestion and I am willing to make further revision to shortcomings. (Page 4, Line 11-23)

(In summary, most existing studies start from enterprise performance and use OLS regression to explore the impact of digital transformation. However, as a factor in enterprises’ independent decisions, digital transformation has strong subjectivity, leading to strong endogeneity. To eliminate its impact, we establish a difference-in-differences model for the research that establishes dual dummy variables for the regression to lower endogeneity. There are a few previous studies on the impact of digital transformation on enterprise core competitiveness that reflect the fundamental survival factor of enterprises. At the same time, previous studies have not studied the enterprises in the industry that would benefit more from the digital transformation from the industry perspective; therefore, we build an evaluation index system of enterprise core competitiveness, analyses the role and mechanism of digital transformation affecting the core competitiveness of enterprises, and proposes policy and practical guidance related to digital transformation to promote the core competitiveness of enterprises.)

6.Comments:Methodology: Model.. I suggest authors here build your main heading on Research and data methodology. Clearly explain the model building process, and what previous studies have used similar models (model testing approach).

Response: Considering the Reviewer’s suggestion, we have changed the position of the description of the model construction process. At the same time, we think that the model testing approach you mentioned is necessary, so we used previous studies to show that it is appropriate to study Digital transformation by using the DID model.(Page 15,16)

(Heckman et al. (1985) were the first to propose the use of DID methods for the assessment of the public policy effect. Since then, there has been a proliferation of research results on DID methods. Zhang, Q (2022) used the DID model and discovered that green innovation output increases significantly through the implementation of corporate digital transformation[38]. Wang, Q (2022) found that digital transformation significantly reduces electricity consumption and intensity, and this electricity-saving effect is achieved through technological optimization and industrial upgrading brought about by digital transformation[39]. Tao Zhang (2022) revealed that the implementation of digital transformation plays a significant role in promoting economic benefits.[40]

There are many more management papers that have used the DID method to research digital transformation, which indicates that DID is suitable for researching this topic.

Therefore, to verify Hypothesis H1, we set the following double difference model:

The core explanatory variable of this model is the interaction item of treat and time. The treat variable is used to delineate the experimental group and the control group. We obtain the degree of digital transformation of the steel industry from the CSMAR database and perform logarithmic processing. During the observation period, if the degree of digitalization is greater than the average value of the sample, the variable takes the value of 1 and 0 otherwise. The time variable is used to divide the time point of the impact of digital transformation. Considering that the Guiding Opinions of the State Council on Actively Promoting the Action of "Internet plus" issued by the State Council in 2015 initiated the development pattern of China's digital economy, we select 2015 as the policy time point. After that year, the value of this variable is 1 and 0 before that year.)

7.Comments:There is no flow in the text. It partly depends on the lack of proofreading but also on the fact that many statements and claims are made without being followed up by a clear and logical discussion. It is especially problematic in the Introduction that brings up a number of findings from different areas without linking them together.

Response: We appreciate it very much for this good suggestion, and we have done it according to your ideas. We linked them together by summarizing their studies and highlighting our study. (Page 4, Line 11-23))

8.Comments:Please make sure your conclusions' section underscores the scientific value-added of your paper, and/or the applicability of your findings/results. Highlight the novelty of your study.

In addition to summarizing the actions taken and results, please strengthen the explanation of their significance. It is recommended to use quantitative reasoning comparing with appropriate benchmarks, especially those stemming from previous work. See the following: How to Improve the Quality and Speed of Green New Product Development? Processes 2019, 7, 443. https://doi.org/10.3390/pr7070443

Response: We appreciate for your valuable comment. We completely rewrote the last paragraph relying on the structure provided by your tenth question and the paper recommended by you. That really helps me a lot, and makes me have a clearer thought of the last paragraph. We really appreciate your suggestion.(Page 32-35)

9.Comments:More importantly, the choice of the variables should be explained in light of the theory and the prior literature on the topic. The arguments are simply relationships and causes very close to the replication of many studies dealing with the same thing.

Response: We totally agree with your suggestion. This paper was based on the resource-based theory. The core of an enterprise's competitive advantage lies in how to obtain heterogeneous resources, that is, resources with high value, scarce, hard to copy and irreplaceable. As a unique heterogeneous resource of an enterprise, Digital transformation can help enterprises improve their core competitiveness and thus gain competitive advantages. And at the same time, two moderating variables: the source of business efficiency and management capability come form the theory of dynamic capability. In the process of enhancing core competitiveness through digital transformation, the level of dynamic capabilities of an enterprise determines the efficiency and effectiveness of the improvement. Higher levels of dynamic capabilities cannot ensure that all strategic decisions of an enterprise are accurate and accurate, however, they can enable enterprises to respond effectively when encountering accidents and errors, and dynamic capabilities have a positive role in improving enterprise competitiveness and performance.

Additionally, as for those arguments, we think the main purpose of that is to create the “gap”, so there indeed has many studies dealing with the same thing, but they aim at enhancing the persuasiveness, displaying those conclusion is not a standalone case. Your concerns are definitely meaningful and making sense, and we have carefully considered it. However, we think maybe the way of our expression is suitable for showing the “gap”. Thanks again for your suggestion, and we are willing to make further modification, if you suggest.

10.Comments:The authors should emphasize the important role of digital technology in green innovation in future research.

Response: Thanks for your suggestion. Green innovation is a hot topic and have a wilder filed to explore. The paper suggested by you provide us the standard of writing, and the topic is attractive and novel, presenting in a very scientific way, which really inspires our research ideas. Thank you again for providing us with such an excellent paper for reference. We believe us next paper will be in this direction.

11.Comments:Please consider this structure for manuscript final part.

-Discussion

-Conclusion

-Managerial Implication

-Practical/Social Implications

-Discussion needs to be a coherent and cohesive set of arguments that take us beyond this study in particular, and help us see the relevance of what authors have proposed. Authors should create an independent “Discussion” section. Author need to contextualize the findings in the literature, and need to be explicit about the added value of your study towards that literature. Also other studies should be cited to increase the theoretical background of each of the method used. Findings should be contextualized in the literature and should be explicit about the added value of the study towards the literature (An adoption-implementation framework of digital green knowledge to improve the performance of digital green innovation practices for industry 5.0, https://doi.org/10.1016/j.jclepro.2022.132608.). Limitations and future research.

As any emprical study that use different approaches I would like to ask to introduce in the Conclusion section at least a paragraph containing the study limitations. I noticed some things in the paper but a synthesis of statements related to how the study is useful (or partially useful, since are required certain further analysis) and helps potential interested readers does not really exist. Maybe in addition to the last section of Conclusion it is beneficial to introduce a section called: Discussion.

Response: Thanks again for your valuable feedback. We have revised the text to address your concerns and hope that it is now clearer. We hope our modifications can improve the quality of the article, and we are also willing to make further modifications based on your feedback.(Page 32-35)

Special thanks for your good comments! Your work has helped us to improve the quality of the paper considerably and has made my research more logical and clearer, especially for the introduction and the conclusion paragraph, which might be the most important part of the paper, we have a clearer logical framework of that, thanks again for your well-thought-out comments, that really helped us a lot!

**Reviewer #2:**

1.Comments:It is a highly fascinating subject. The concept is emerging and might be helpful in today's actual world, specifically in the supply chain and technological transformations field. Therefore, I recommend this article for further process based on the paper's relevance, originality, need, and suitability for the journal. The introduction is skillfully crafted. However, the author may guarantee the order in which the concepts are explained and presented.

Response: We deeply appreciate the reviewer’s suggestion. According to the reviewer’s comment, We have added an explanation about Digital transformation in the first paragraph, and revised the original explanation, hoping that this can enhance the quality of this paper.

2.Comments:The literature review is skillfully prepared and effectively divides various topics with appropriate titles. Every hypothesis development is discussed in the literature, and it is a good idea to describe the theoretical framework after presenting the image. Similar to how the approach is taught, it is presented logically but still needs to be well integrated into the paper.

Response: We are extremely grateful to reviewer for pointing out this problem. We have summarized the limitations of these studies and highlighted the highlights of our research, hoping to stand out the 'gap' of our research.Additionally, before describing the theoretical framework of the article, we sketch out its framework in pictures.(Fig 1).

3.Comments:In addition, I wonder if the authors should revise their literature and incorporate more pertinent literature. A few of the pertinent articles I am providing to the author that must be incorporated into this study are listed below:

• Khan, S.A.R., Ahmad, Z., Sheikh, A.A. and Yu, Z. (2023), "Green technology adoption paving the way toward sustainable performance in circular economy: a case of Pakistani small and medium enterprises", International Journal of Innovation Science, Vol. ahead-of-print No. ahead-of-print. https://doi.org/10.1108/IJIS-10-2022-0199

• Rehman Khan SA, Ahmad Z, Sheikh AA, Yu Z. Digital transformation, smart technologies, and eco-innovation are paving the way toward sustainable supply chain performance. Science Progress. 2022;105(4). doi:10.1177/00368504221145648

• Khan, S. A. R., Tabish, M., & Zhang, Y. (2023). Embracement of industry 4.0 and sustainable supply chain practices under the shadow of practice-based view theory: ensuring environmental sustainability in corporate sector. Journal of Cleaner Production, 398, 136609.

Response: Thank you for your recommendation, the topics of these papers are very innovative and very relevant in the current environment, they can better help companies to carry out green innovation and thus promote sustainable development of the environment, which are very important in the current polluted society, at the same time the research methods of these papers are very innovative and the research ideas are very rigorous, they have a huge reference value for me, and also It has inspired me to think about conducting similar research, and our next paper is likely to be in this direction, so thank you again for recommending such excellent papers to us!

4.Comments:Last but not least, ensure all references are accurate and include all relevant information, such as the volume, issue, and page numbers. Well, the study complied with the requirements set forth by the esteemed publication by providing all pertinent data and by covering all essential topics. To ensure optimum refinement, the author should double-check each line while considering the sample article from a prominent publication.

Response: Thank you for the suggestion After careful proofreading, we have revised the reference format to take into account the journal's requirements.

Thank you for your valuable comments, which have helped improve this thesis's quality and pointed me in the right direction for future research. The papers you have provided are indeed theoretically solid, and logically clear and have a brief and straightforward thesis statement, and they have provided me with a good reference template, so thank you again for your help!
